# Tuning Properties of Polyelectrolyte-Surfactant Associates in Two-Phase Microfluidic Flows

**DOI:** 10.3390/polym14245480

**Published:** 2022-12-14

**Authors:** Artem Bezrukov, Yury Galyametdinov

**Affiliations:** Department of Physical and Colloid Chemistry, Kazan National Research Technological University, 420015 Kazan, Russia

**Keywords:** microfluidics, polyelectrolyte, surfactant, association, aggregation, emulsion, microdroplets

## Abstract

This work focuses on identifying and prioritizing factors that allow control of the properties of polyelectrolyte-surfactant complexes in two-phase microfluidic confinement and provide advantages over synthesis of such complexes in macroscopic conditions. We characterize the impact of polymer and surfactant aqueous flow conditions on the formation of microscale droplets and fluid threads in the presence of an immiscible organic solvent. We perform an experimental and selected numerical analysis of fast supramolecular reactions in droplets and threads. The work offers a quantitative control over properties of polyelectrolyte-surfactant complexes produced in two-phase confinement by varying capillary numbers and the ratio of aqueous and organic flowrates. We propose a combined thread-droplet mode to synthesize polyelectrolyte-surfactant complexes. This mode allows the production of complexes in a broader size range of R ≈ 70–200 nm, as compared with synthesis in macroscopic conditions and the respective sizes R ≈ 100–120 nm. Due to a minimized impact of undesirable post-chip reactions and ordered microfluidic confinement conditions, the dispersity of microfluidic aggregates (PDI = 0.2–0.25) is lower than that of their analogs synthesized in bulk (PDI = 0.3–0.4). The proposed approach can be used for tailored synthesis of target drug delivery polyelectrolyte-surfactant systems in lab-on-chip devices for biomedical applications.

## 1. Introduction

Interactions between oppositely charged polyelectrolytes and surfactants have attracted a sustainable, fundamental, and applied research interest [1,2,3,4,5]. Self-organization in polyelectrolyte-surfactant solutions causes the formation of functional nanoscale systems [2,6], which are used in nanotechnology [7,8] and medicine [4,9]. Polyelectrolyte-surfactant complexes are demanded for template synthesis of materials [10,11], target drug delivery systems [4,7,12,13], and fabrication of soft matter with smart stimuli-responsive capabilities [5,14].

Microfluidics provides new options by which to control synthesis [15,16] and properties [17,18] of polyelectrolyte-surfactant complexes, as compared with macroscopic conditions. Microfluidic confinement in the range of dozens or hundreds of micrometers creates a non-equilibrium environment inside microchannels [19], which allows bottom-up engineering of soft matter in ordered diffusion-controlled reactive flows [20,21,22,23] or emulsion droplets [24,25,26]. Microfluidic synthesis produces nanoparticles with a broader size range and less dispersity than in macroscopic conditions [18,21,23,26]. Microfluidic devices expand synthesis and application horizons of functional polyelectrolyte systems, especially for diagnostics [27,28] and drug delivery medicine [22,25,29].

Two-phase microfluidic flows, represented by immiscible aqueous and organic solvent media, form even more confined zones inside microchannels, such as focused threads [30,31] or picoliter-size droplets [32,33,34]. In such microzones, reactions occur in ordered diffusion or fast mixing conditions in an aqueous phase [35]. Microfluidic droplets, therefore, precisely control reaction processes [35,36] and offer optimized conditions for synthesis of functional nanoscale [18,26] or microscale [37,38,39] polymer systems.

Microfluidic devices are capable of generating a variety of two-phase systems: droplets of various sizes, stable central or side threads of various widths, or threads that further break-up into droplets [30,34,40,41]. Combining different two-phase systems in a single microfluidic device may offer extremely variable conditions for synthesis or modification of polyelectrolyte-surfactant complexes through controlling the parameters of droplets and threads by flow and fluid properties [30,42].

Multiple publications have discussed the behavior of polymer and surfactant systems in two-phase microfluidics. These publications, however, have mostly focused on processes in uniform-sized microfluidic droplets [18,35,38,43,44,45] and have not paid thorough attention to reactive media in variable size droplets, threads, or combined thread-droplet systems. In this regard, a detailed analysis of polyelectrolyte-surfactant complexation in variable two-phase microfluidic flows may provide new approaches to the tuning properties of functional polyelectrolyte systems.

The goal of this work was to identify and prioritize factors that govern polyelectrolyte-surfactant complexation in two-phase microfluidic flows and control the properties of associates. We studied the impacts of polyelectrolyte and surfactant additives on formation of microscale aqueous droplets and threads. We performed an experimental analysis of complexation in variable size droplets and applied a combined experimental and numerical approach to characterize reaction conditions in aqueous threads of various widths. This work proposes a new thread-droplet method for synthesis of polyelectrolyte-surfactant associates. This method minimizes residual after-chip reactions and produces complexes with a broader size range and reduced dispersity, as compared with synthesis in macroscopic conditions.

## 2. Materials and Methods

### 2.1. Materials

Polydiallyldimethylammonium chloride (PDADMAC) was purchased from Sigma Aldrich, St. Loius, MO, USA. The polymer was sold as a viscous liquid (20% aqueous solution) and was used as received. Sodium dodecyl sulphate (SDS) was purchased from BDH Limited, Poole, England, and was used as received. Surfactant was sold as a powder.

We selected these materials as model substances because the macroscopic association and phase behavior of such systems and their analogs is well-characterized [6,46,47,48]. We found SDS diffusion coefficients and interfacial tension data in the literature [49,50] and estimate association rate constants of similar polyelectrolyte-surfactant systems [51,52].

Deionized water (18.2 MΩ-cm) was used for all aqueous polyelectrolyte and surfactant solutions. Before preparing solutions, water was filtered by 0.2 µm Millipore PTFE filters. Although the PTFE filter is hydrophobic, its pores provide good penetration of water through the membrane. Hexadecane (Reachem, Moscow, Russia, analytic grade purity) was used as an organic solvent in all the experiments.

Polydimethylsiloxane (PDMS) Sylgard 184 was purchased from Dow Corning (Midland, MI, USA) and used to fabricate microfluidic devices. It comes as a two-part elastomer kit (the pre-polymer and curing agent). SU-8 3050 photoresist (Microchem Corp., Westborough, MA, USA) was used to produce a mold for microfluidic chips.

### 2.2. Solutions

For both macroscopic and microfluidic experiments, bulk samples of 1 g/L PDADMAC were produced from its initial solution and allowed to dissolve overnight. An amount of 50 mmol/L KCl (Reachem, Moscow, Russia) was used as a background electrolyte. The concentration of monomers in 1 g/L PDADMAC solution was 6.1 × 10^−3^ mol/L. Bulk samples of 6.1 × 10^−3^ mol/L SDS were produced by dissolving dry surfactant in deionized water.

For macroscopic experiments, PDADMAC-SDS solutions were combined in different ratios by volume to provide polymer-surfactant compositions with the surfactant-to-polymer molar ratios that varied in the 0–1.0 range.

For microfluidic experiments, hexadecane, as well as PDADMAC and SDS solutions, were infused into microfluidic devices by Shenchen ISPLab01 syringe pumps. The flowrates of polymer and surfactant aqueous solutions and the organic solvent were varied in the range of 0.25–15 µL/min. To provide the same hydraulic path of fluids to all the inlets, we used PTFE tubes of identical lengths (10 cm) and internal diameters that fit the same needle tips inserted into microchip outputs (20 G-type needles, 0.9 mm diameter). These tubes were connected to identical 1 mL syringes installed into syringe pumps.

For better visualization of polyelectrolyte and surfactant flows during image recording, 5–7 droplets of phenolphthalein in 0.01 M KOH (Reachem, Moscow, Russia,) and 5 wt.% iodine solution in ethanol (MosFarma, Moscow, Russia) were added to 5 mL of surfactant and polyelectrolyte solutions, respectively. These dyes provided high-contrast bright magenta and amber colors to these solutions, so we could distinguish microfluidic droplets and coflowing threads and track mixing of reactive solutions in real time. No dyes were added to reactive solutions, which were used for producing samples of PDADMAC-SDS complexes for further size characterization.

### 2.3. Methods

Hydrodynamic diameters and dispersities of PDADMAC macromolecules and PDADMAC-SDS complexes synthesized in macroscopic and microfluidic solutions were measured using a Malvern Zetasizer Nano ZS light scattering system (Malvern, Worcestershire, United Kingdom). PDI is the polydispersity index of aggregates taken from the Malvern Zetasizer Nano software report. The PDI range is [0; 1]. PDI values are lower for monodisperse particles and higher for polydisperse particles. All the DLS measurements were repeated at least three times to obtain reproducible results.

To take the samples from microchips, we collected them in glass vials through the PTFE tube inserted into the microchip output, and then took the upper aqueous phase by 1 mL syringe. For further DLS analysis of the aqueous phase, we used Malvern ZEN0040 micro-cuvettes, which require a minimal analytical volume of 40 µL. Such cuvettes allowed for fast sampling of microfluidic polymer-surfactant complexes, even at low flowrates. In this work, we report sizes of complexes, which correspond to the maximum of the curve plotted in the intensity-size coordinates. Intensity-average distribution is highly sensitive to sizes of particles (I~R^6^, where I is the scattered light intensity and R is particle radius), so it is expected to be more indicative for tracking changes in sizes of PDADMAC-SDS aggregates synthesized in different chip operation modes.

Images were recorded on a Levenhuk D320 optical microscope (Levenhuk, Tampa, FL, USA). Microchannels were imaged at 10× magnification using a Levenhuk M1400 Plus camera with a resolution of 0.27 µm/pixel.

Convection-diffusion-reaction equations for reacting polyelectrolyte-surfactant flows in aqueous threads were solved using Matlab 2021a software with Partial Differential Equations Toolbox. Diffusion coefficients of SDS ions were obtained from [49]. For simulations, the value of the polyelectrolyte-surfactant association rate constant was set to 10^4^ L·mol^−1^·s^−1^.

### 2.4. Device Fabrication

Microfluidic devices were fabricated using standard photolithography techniques [53]. SU-8 photoresist and a transparency photomask with the negative image of a microchip were used to produce a 100 µm thick mold of microfluidic chips on top of a 3-inch silicon wafer. Sylgard 184 PDMS pre-polymer was mixed with a curing agent, poured over the mold, and allowed to cure for 4 h in a 60 °C oven. Once cured, PDMS was peeled off the mold and bonded to a flat PDMS slab via plasma treatment. The PDMS device was then heated in an oven at 180 °C for 1 h to finalize bonding of two polymer layers.

## 3. Results and Discussion

### 3.1. Characterization of PDADMAC-SDS Complexes Synthesized in Macroscopic Conditions

Interactions of oppositely charged polyelectrolytes and surfactants in macroscopic conditions have been discussed in detail in the literature [1,2,3,6]. Depending on the ratio of components, various complexation scenarios result in the formation of nanoscale associates that may differ in size, conformations of macrochains, colloidal stability, and the resulting phase behavior. In our previous works, we characterized PDADMAC-SDS mixtures and similar systems of oppositely charged polyelectrolytes and surfactants in bulk [54] and single-phase microfluidic confinement [55]. Before studying PDADMAC-SDS complexation in two-phase microfluidic systems, we tested the properties of the purchased polymer and surfactant samples in macroscopic aqueous solutions.

**Figure 1:**
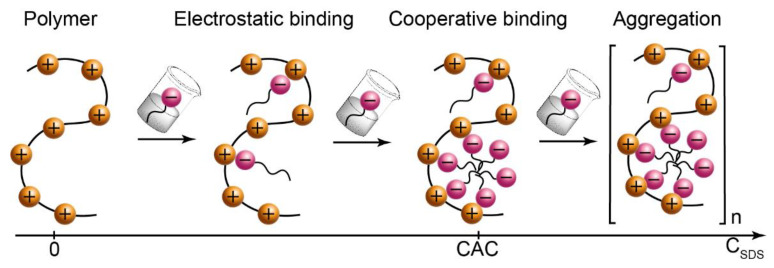
Binding surfactant ions by polyelectrolyte macrochains expected for the PDADMAC-SDS solutions at various concentrations of added surfactant. CAC is the critical association concentration.

Figure 1 demonstrates a scheme that is typical for binding surfactant ions by oppositely charged polyelectrolyte macromolecules [6].

Binding sites at polyelectrolyte macromolecules are represented by quaternary nitrogen in its monomer units. They reversibly bind SDS ions by the electrostatic mechanism at their low concentrations. When the concentration of added surfactant reaches its critical association concentration or CAC (similar to critical micellization concentration or CMC in individual surfactant solutions), micelle-like aggregates form along macrochains; this was confirmed for the PDADMAC-SDS pair [47]. CAC is generally reported to be 1–2 orders of magnitude lower than CMC [1,6].

When the concentration of added surfactant exceeds CAC, complexation involves the hydrophobic effect and polymer macrochains bind to nearly all available surfactant ions by the cooperative binding mechanism [6,54]. Neutralization of macroions reduces their colloidal stability and initiates aggregation and possible precipitation. Aggregates may vary in size and dispersity depending on the ratio of components, synthesis conditions, and other factors.

Characterization of PDADMAC-SDS systems by dynamic light scattering confirm that they generally follow the complexation scenario shown in Figure 1. The amount of added surfactant can be conveniently characterized by the surfactant/polymer ratio Z. Initial PDADMAC solutions (Z = 0) are transparent and contain particles with the hydrodynamic radius R ≈ 15–20 nm, which represent individual polymer macromolecules. Slight amounts of added surfactant (Z = 0.01–0.05) do not exert a considerable impact on the size of particles and transparency of solutions, indicating that forming complexes do not aggregate, although both electrostatic and cooperative binding mechanisms are possible. In the range Z = 0.05–0.5, solutions become turbid and particles grow in size from R ≈ 15 nm to R ≈ 150–200 nm before the system undergoes precipitation. Larger particles indicate that polymer macrochains with bound surfactant ions aggregate into larger particles. The polydispersity index of all the particles is PDI ≈ 0.3–0.4.

According to the results of macroscopic characterization, we performed further microfluidic experiments with initial SDS and PDADMAC solutions that provide the ratio Z = 0.2. At this ratio, PDADMAC and SDS form soft matter particles with the hydrodynamic radius R ≈ 100–120 nm. The system is expected to undergo cooperative binding and aggregation without undesirable precipitation that may clog microchannels. Microfluidic droplets and threads are supposed to create variable concentration gradients or mixing conditions for polymer and surfactant reactive flows, and, therefore, synthesize a variety of PDADMAC-SDS aggregates from the same precursor solutions.

### 3.2. Determining Flow Conditions for Performing PDADMAC-SDS Association in Microfluidic Droplets and Fluid Threads

**Figure 2:**
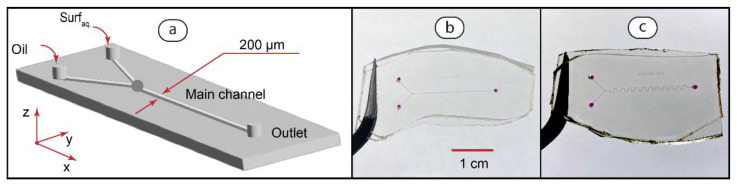
Design of Y-type microfluidic chips for testing the formation of droplets and threads by individual polymer and surfactant solutions (**a**) and the fabricated devices with the straight (**b**) and serpentine (**c**) main channel geometries. Channels are filled with dye (phenolphthalein in 0.01 M KOH) for visualization.

In the next stage of this work, we studied the impact of individual polyelectrolyte and surfactant solutions on the formation of two-phase microflows. Figure 2 represents the design and photos of microfluidic mixers fabricated for this stage. The main channels (straight and serpentine) are the same as in the microfluidic reactors used in further experiments.

A common approach to characterizing physicochemical processes in microfluidic confinement is the introduction of similarity criteria [56]. The governing dimensionless parameters in production of two-phase microfluidic flows include the capillary number:

(1) $$\mathrm{Ca}=\frac{\mu_{\mathrm{oil}}U}{\gamma }$$

where μ_oil_ is the viscosity of continuous (oil) phase, U is the flow velocity in the main channel, and γ is the interfacial tension between aqueous and oil phases. These parameters also include the flow rate ratio:

(2) $$\varphi =\frac{Q_{\mathrm{aq}}}{Q_{\mathrm{oil}}}$$

where Q_aq_ and Q_oil_ are the flowrates of aqueous and oil phases, respectively, and the viscosity ratio:

(3) $$\sigma =\frac{\mu_{\mathrm{aq}}}{\mu_{\mathrm{oil}}}$$

where μ_aq_ and μ_oil_ are the viscosities of aqueous and oil phases, respectively.

Capillary number is a key parameter that evaluates transitions between different modes of two-phase microflows. At low capillary numbers, Ca < 0.01, droplets are generated inside the main channel. At high capillary numbers (Ca > 0.1), the fluid interface does not readily break into droplets, and coflowing fluid threads form in the entire main channel at any flow rate ratios. At intermediate capillary numbers, such threads break at a certain distance after the junction of inlets. The sizes of droplets and widths of threads can be controlled by the flow rate ratio. The droplet length is proportional to the flow rate ratio φ, while the thread width is proportional to φ^1/2^ [30].

The viscosity of low concentration polyelectrolyte-surfactant solutions approaches that of a pure solvent [6], so we do not expect considerable changes in the σ = μ_aq_/μ_oil_ parameter in reactive diluted polymer-surfactant systems.

A key property of surfactants that directly affects microfluidic droplet generation is that they reduce interfacial tension. According to the literature [50], the introduction of SDS to the aqueous phase in amounts used in our experiments (1.2 × 10^−3^ mol/L) reduces interfacial tension between the aqueous phase and hexadecane to approximately 10 mN/m. The resulting increase in capillary number brings us closer to its intermediate range of Ca = 0.01–0.1 at lower flowrates. With such a combination of factors, we can both vary reaction conditions by generating fluid droplets or threads, and be more flexible with the residence time of the reagents in a microchip. In turn, the residence time is an important parameter that controls the size of forming particles [18,36,57].

**Figure 3:**
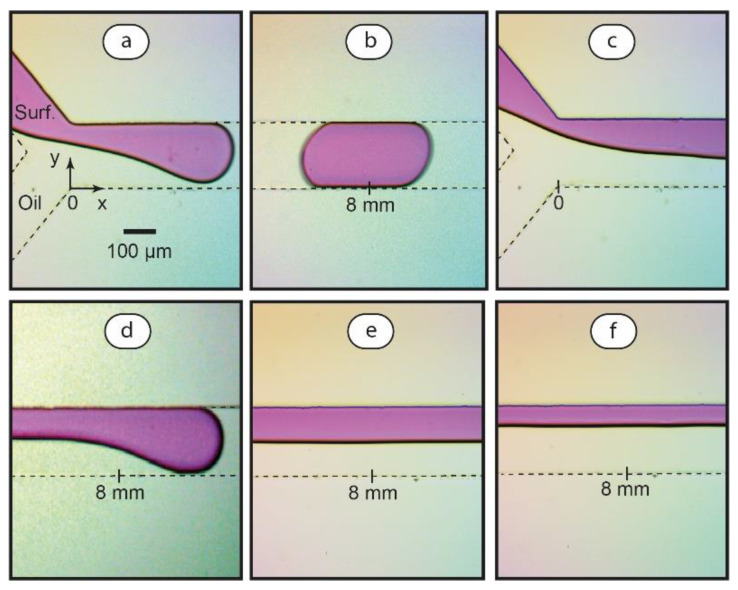
Optical microscopy images of aqueous SDS (1.2 × 10^−3^ mol/L) and hexadecane flows: droplet formation (**a**) and break-up (**b**) at Ca ≈ 0.005 and φ = 0.1; thread generation (**c**) and break-up (**d**) at Ca ≈ 0.03 and φ = 1; stable threads at Ca ≈ 0.05, φ = 1 (**e**) and Ca ≈ 0.05, φ = 0.3 (**f**). Dye (phenolphthalein + 0.01 M KOH) was added to the surfactant solution for visualization. Dashed lines mark microchannel boundaries.

We evaluated capillary numbers for the aqueous SDS—hexadecane system and performed microfluidic experiments in conditions near the intermediate range of Ca, achieved within flowrates of 3–30 μL/min. Figure 3 summarizes the results.

As we can see in Figure 3a,b, the droplet generation mode is observed at Ca ≈ 0.005. The droplet generation process is typical for that reported in the literature [30]: the interface fully obstructs the main channel (Figure 3a) and then finally pinches off (Figure 3b).

In the droplet generation mode, the size of droplets is known to depend on microchannel geometry and flowrates of aqueous and oil phases [42]. We characterized sizes of droplets in varied flow conditions for the same main channel geometry (200 µL width and 100 µL height). According to the experiments, longer droplets are generated at higher flow rate ratios φ provided that the resulting capillary number Ca < 0.01. These droplets fully obstruct the rectangular main channel. It approximately evaluates the volumes of these droplets as equivalent rectangular parallelepipeds: V ≈ l WH, where l is droplet length, W is microchannel width, and H is microchannel height. In the range of φ = 0.05–0.2, the length of droplets grows from ≈0.5 mm to ≈3–4 mm, while their volume grows from ≈10 picoliters to ≈60–80 picoliters, respectively. The growth of droplets at higher flow ratios agrees with the literature data [30].

A combined thread-droplet mode is observed in the flows if Ca is increased to its intermediate level Ca > 0.01 (Figure 3c,d). At Ca ≈ 0.03, a coflowing fluid thread forms at the intersection of the input channels. The thread breaks up into droplets at a distance from the intersection point. This distance grows at higher flowrates (and, therefore, capillary numbers). At Ca ≈ 0.05, the channel is predominantly occupied by coflowing threads (Figure 3e,f) and the thread break-up occurs near the main channel outlet. Thinner threads of the SDS phase form at lower flow rate ratios φ. At higher values of Ca, threads remain stable along the entire channel.

In surfactant flows, therefore, aqueous threads start to form and elongate in the intermediate capillary number range. In such conditions, a flowrate ratio φ becomes a more convenient control parameter for two-phase flows than the capillary number. According to the literature [30], a criterion for the formation of stable threads is:

aφσ > 1(4)

where a = w/h is the width-to-height ratio of the main channel. For our microchip geometries and solvents (a = 2, σ ≈ 3), the transition to threads is evaluated to occur at the flow rate ratio φ > 0.2. This agrees with our experiments. Therefore, we can control transitions between droplets and threads in a medium range of Ca (flowrates 3–30 μL/min) by mostly varying the ratio φ.

The experiments shown in Figure 3 were repeated for two-phase pairs, represented by water + hexadecane, aqueous PDADMAC + hexadecane, and pre-synthesized PDADMAC-SDS complex (Z = 0.2) + hexadecane.

With the solution of polyelectrolyte-surfactant complex, we were able to generate the same two-phase flow modes as those demonstrated in Figure 3 in the range of flow rates about 3–30 μL/min. The results revealed no considerable differences between flowrates required to produce droplets, threads, or perform transitions between them for PDADMAC-SDS complexes, as compared with individual SDS solutions. This may be associated with the fact that the presence of surfactants in polyelectrolyte-surfactant solutions may provide a similar reduction in interfacial tension [6].

For aqueous PDADMAC + hexadecane and water + hexadecane systems, we were unable to achieve a transition to threads at reasonable flowrates below 100 μL/min. With the absence of surfactant, therefore, the capillary number remains high and the droplet generation mode prevails.

Microfluidic chips with serpentine channels can be favorable devices for performing reactions in droplets because they provide fast mixing capabilities [36]. The experiments shown in Figure 3 were also performed in microchips with serpentine main channels, as shown in Figure 2c. The results were almost identical to those obtained in straight channels and agree with the literature data [30], reporting that capillary number and flow rate ratios are controlling parameters for two-phase flows in microchips with various geometries.

Droplets and threads, as shown in Figure 3, represent potential confined reaction zones for the PDADMAC-SDS pair. Such reaction zones are expected to be considerably different in their properties and capable of providing synergy in tailoring polyelectrolyte-surfactant complexation. The next stages of this work, therefore, focused on the synthesis of PDADMAC-SDS associates in microfluidic droplets and threads, and combined droplet-thread flows.

### 3.3. Synthesis of PDADMAC-SDS Complexes in the Droplet Mode: Impact of Droplet Size and Microchannel Geometry

**Figure 4:**
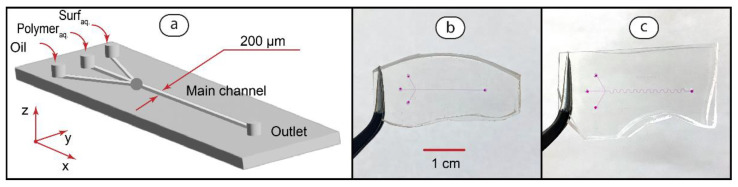
Design of W-type microfluidic chips for performing polyelectrolyte-surfactant reaction in two-phase flows (**a**) and the fabricated devices with the straight (**b**) and serpentine (**c**) main channel geometries. Channels are filled with dye (phenolphthalein in 0.01 M KOH) for visualization.

To synthesize PDADMAC-SDS complexes in two-phase microfluidic flows, we re-designed the chips shown in Figure 2 by adding an additional inlet for the polymer solution. Figure 4 demonstrates the design and photos of microfluidic reactors fabricated for this stage.

In these chips, polymer and surfactant solutions are fed through neighboring inlets to make a reaction mixture. The flow of hexadecane is fed through the third inlet to confine this mixture in droplets or threads. Straight (Figure 4b) and serpentine (Figure 4c) main channels are identical to those shown in Figure 2.

A key difference between reaction conditions in microfluidic droplets and straight flows is known to be the mixing of the reagents. In mobile microdroplets, reactive solutions undergo intensive convective mixing [35,36]. In parallel laminar reactive flows, mixing is diffusion-controlled and colloidal particles, such as polymers and surfactants, do not completely mix in microchannels [56].

**Figure 5:**
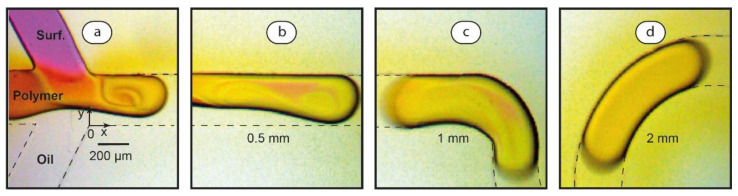
Optical microscopy images of SDS, PDADMAC, and hexadecane flows: droplet formation (**a**) and break-up (**b**), continuing mixing of solutions (**c**) and complete mixing (**d**). U = 30 mm/s, Ca ≈ 0.005, and φ = 0.1. Dyes (phenolphthalein + 0.01 M KOH and iodine) were added to surfactant and polymer solutions, respectively, for visualization. Dashed lines mark microchannel boundaries.

To visualize mixing in PDADMAC and SDS microscale flows, we added dyes to their solutions and pumped them through microchips, as shown in Figure 4. We started with serpentine chips, which have been reported to provide a more intensive convective mixing of solutions due to their curved geometry [36]. Figure 5 summarizes the results.

Figure 5 visualizes intensive convective mixing of polymer and surfactant solutions in droplets. A forming droplet (Figure 5a) contains a vortex of reactive solutions visualized by dyes of magenta and amber colors. When this droplet pinches off (Figure 5b), intensive mixing continues and the vortex pattern of convection is clearly distinguishable. At the 1 mm distance from the junction point, however, (Figure 5c) patterns of different colors become harder to recognize, which indicates the interpenetration of polymer and surfactant reactive flows. Finally, the droplets obtain a uniform and constant color 3 mm after the junction of the inlets (Figure 5d), demonstrating that mixing is complete. Increasing volume of droplets at higher values of the flowrate ratio φ resulted in a slight increase of the mixing distance (≈2.5 mm for φ = 0.2).

Figure 5 evaluates the time of mixing in droplets. For the flow velocity of U = 30 mm/s, mixing the reagents is complete when the droplet reaches the L = 2 mm distance point, t_mix_ = L/U ≈ 60–70 ms. We can compare this value to the characteristic time of the polymer-surfactant reaction. For the approximate value of the rate constant k = 10^4^ and the initial concentration of surfactant C^0^ = 1.2 × 10^−3^ mol/L, the characteristic time of reaction is t_r_ = 1/(kC^0^) ≈ 80 ms. We can see that t_mix_ and t_r_ are comparable for serpentine chips.

The same experiments were performed in microfluidic chips with linear main channels (Figure 4b). Complete mixing in the linear channel was achieved at larger distances from the junction point (approximately 6–7 mm), which increased at higher flowrate ratios (≈9–10 mm for φ = 0.2). For the studied microchips, therefore, a longer mixing time (≈200 ms for φ = 0.1) is required for solutions in droplets in linear channels than in serpentine ones. These results agree with the literature [36], reporting that curved geometries facilitate convective mixing in microdroplets.

Thus, characteristic times of fast polyelectrolyte-surfactant complexation reactions can be comparable with mixing times of solutions in microfluidic droplets. The mixing time depends on the main channel geometry and flow conditions. In turn, these factors may influence the complexation reaction and characteristics of forming associates, such as their size and dispersity.

**Figure 6:**
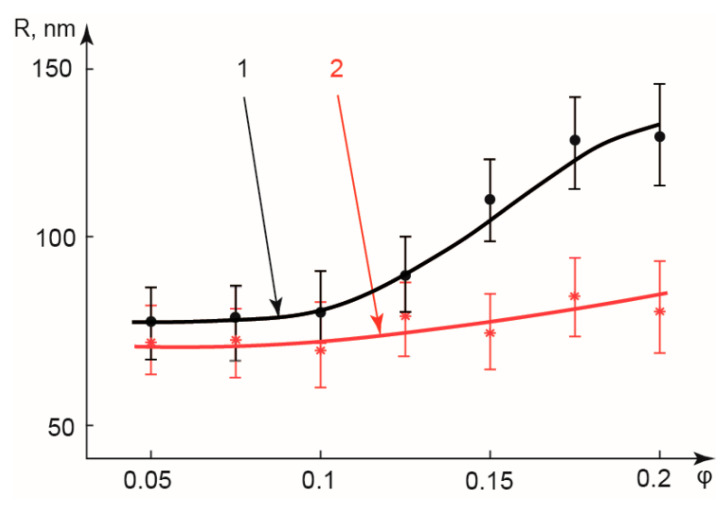
Hydrodynamic radii of PDADMAC-SDS complexes synthesized in microfluidic droplets at various flowrate ratios φ: 1—linear main channel; 2—serpentine main channel. Ca ≈ 0.005.

To verify this assumption, we performed experiments with PDADMAC and SDS solutions (without dyes) in conditions shown in Figure 5. We took samples of PDADMAC-SDS solutions from the outlets of linear and serpentine chips at various flowrates ratios φ and characterized them by DLS. Figure 6 demonstrates the results.

The experiments summarized in Figure 6 focus on the characterization of nanoscale particles that emerge as the result of PDADMAC-SDS complexation in microfluidic confinement. We studied the impact of flow conditions on hydrodynamic radii and dispersities of the aggregate’s polyelectrolyte-surfactant complexes. The sizes of particles vary in the range of 70–120 nm. PDADMAC-SDS complexation in smaller droplets (φ = 0.05–0.1) produces smaller aggregates (R ≈ 70–80 nm) than in macroscopic conditions (R ≈ 100–120 nm) in both straight and serpentine channels. Dispersities of microfluidic complexes are lower (PDI_micro_ = 0.25–0.3) than those of their counterparts produced in macroscopic conditions (PDI_macro_ = 0.3–0.4). These results agree with other works [18] that have also reported the formation of smaller sized and less dispersed polymer-based nanocomplexes in microfluidic confinement, and may be associated with more uniform reaction conditions in droplets provided after rapid mixing.

At higher flow rate ratios (φ = 0.15–0.2), there are no considerable differences in the size characteristics of aggregates produced in curved channels. In linear channels, however, the size and dispersity of PDADMAC-SDS associates increases and gradually approaches that of complexes synthesized in macroscopic conditions. A possible reason for this effect is less intensive mixing in linear droplets. A non-uniform concentration field exists longer compared with the characteristic time of reaction. The resulting non-uniform binding conditions may produce complexes of a broader dispersity, which are similar to those synthesized in bulk.

Thus, PDADMAC-SDS complexation in microfluidic droplets produced aggregates of a nanoscale radius and lower dispersities than in bulk from the same precursor solutions. In the next stage of this work, we tested if this range could be expanded by performing complexation reactions in linear two-phase threads.

### 3.4. Synthesis of PDADMAC-SDS Complexes in the Thread Mode: Impact of Thread Width and Thread Break-Up

**Figure 7:**
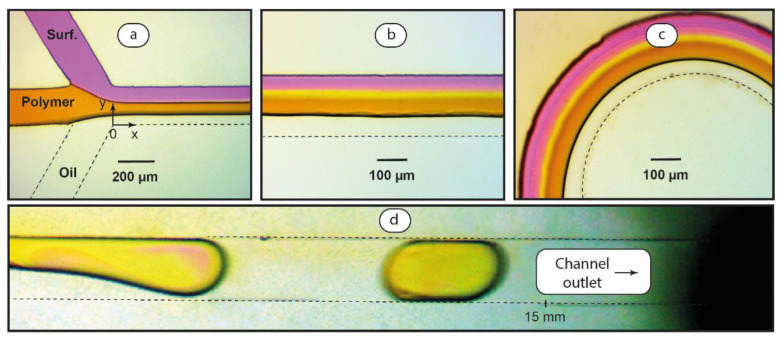
Optical microscopy images of SDS, PDADMAC, and hexadecane flows: linear thread formation (**a**), stable thread in the serpentine channel (**b**) and linear channel (**c**), and thread break-up and mixing of reagents (**d**) near the main channel outlet. Ca ≈ 0.05, φ = 3. Dyes (phenolphthalein + 0.01 M KOH and iodine) were added to surfactant and polymer solutions, respectively, for visualization. Dashed lines mark microchannel boundaries.

Synthesis of polyelectrolyte-surfactant complexes in single-phase laminar microfluidic flows is described in our previous work [58] and in the literature [17,59,60]. The authors report the impact of multiple factors, such as flow width and residence time, on the properties of forming associates. To apply these factors to two-phase microfluidic systems, we confined PDADMAC and SDS solutions in aqueous threads of water and oil flows in microchips. Figure 7 shows such confined stable reactive threads in both linear and serpentine microchannel geometries.

We can see in Figure 7 that polymer and surfactant flows are parallel in the aqueous thread. Starting from the junction point (Figure 7a), no convective mixing is observed compared to the droplet mode shown in Figure 5. This thread mode is stable along the entire main channel in both linear (Figure 7b) and curved (Figure 7c) geometries. A light middle line between magenta and amber threads represents interpenetrating molecules of dyes. It visualizes a slow diffusive mixing, which usually occurs in parallel microfluidic flows [56].

Finally, at lower flow rates, the resulting lower capillary number (Ca < 0.05) threads decompose into droplets (Figure 7d). By varying flow rates at low Ca values, we can achieve decomposition near the main channel end (Figure 7d) or closer to the junction point. At higher capillary numbers, Ca > 0.05 and/or larger flow rate ratios φ > 1, threads occupy entire linear or serpentine channels and do not break-up.

Figure 5 and Figure 7, therefore, show a complete picture of technology that can be used to control polyelectrolyte-surfactant association in two-phase flows. We can generate droplets (Figure 5), where polymer and surfactant solutions rapidly mix and a uniform reaction mixture emerges in a confined droplet reactor. Another option is to generate continuous threads of polymer-surfactant flows where they interact by diffusion (Figure 7a–c). Finally, we can combine a diffusion-controlled reaction in threads and a subsequent rapid mixing in droplets (Figure 7d), and control the thread length by flow conditions.

The experiments with PDADMAC and SDS solutions (without dyes) were performed in conditions shown in Figure 7 for continuous threads first. We took samples from the outlets of linear and serpentine chips and characterized the aqueous phase by DLS. Sizes and dispersities of complexes synthesized in stable threads were similar to those synthesized in macroscopic conditions. Microchannel geometry or flowrates did not exert a considerable impact on size characteristics of the complexes. Such results may be associated with the fact that only a small part of polymer and surfactant particles react in the main channel in slow diffusive mixing conditions. The reaction completes in macroscopic conditions when the reagents leave the microchip, in the same way as we demonstrated earlier for single-phase polymer-surfactant microflows.

**Figure 8:**
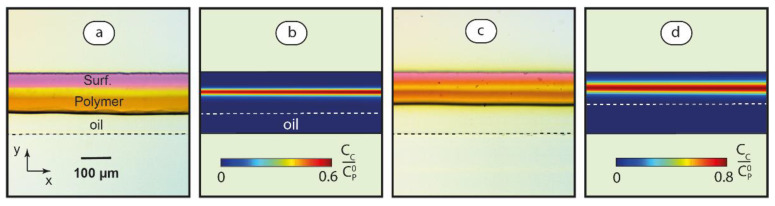
Optical microscopy images and numerical simulation results for PDADMAC-SDS association in linear threads at Ca ≈ 0.05 and different flow rate ratios: φ = 3 (**a**,**b**) and φ = 1 (**c**,**d**). $C_{C}$ is the concentration of surfactant ions bound to macrochain binding sites, $\;C_{P}^{0}$ is the total concentration of macrochain binding sites. (phenolphthalein + 0.01 M KOH and iodine) were added to surfactant and polymer solutions, respectively, for visualization. Dashed lines mark microchannel boundaries.

To verify these assumptions, we performed numerical simulations of PDADMAC-SDS complexation in linear threads of two-phase flows according to the model developed in our previous work [58] for single-phase polymer-surfactant flows. The details regarding customized governing equations, boundary conditions [56], and the Matlab script are provided in Supplementary Materials and Figure S1. Figure 8 demonstrates the concentration distribution of the complexation product in aqueous threads of two-phase microflows.

Under the conditions shown in Figure 8, the threads are stable along the entire main channel. Figure 8a represents a ~150 μm wide aqueous reaction zone. The respective numerical modeling (Figure 8b) reveals a narrow central line of the complexation product with a high local concentration of bound surfactant ions. In a narrower thread of ~100 μm (Figure 8c), diffusive mixing is more intensive, as we can see from less contrasting magenta and amber colors of dyes. Numerical modeling of PDADMAC-SDS solutions (Figure 8d) shows a higher product concentration in a broader reaction zone. It agrees with the literature data on diffusion-controlled reactions in linear microchannel flows, which predict that the mixing rate is inversely proportional to the square width of the flow [56,61].

Following a basic assumption that nanoscale complexation processes are not significantly different at microscale and in bulk, we can expect intensive cooperative binding processes in the reaction front zones, as shown in Figure 8. Polymer-surfactant complexes are less mobile than individual macromolecules, so they are supposed to cooperatively bind to nearly all available surfactant ions diffusing to the reaction zone, and then aggregate and grow in size. We can expect a more intensive complexation and aggregation in broader reaction zones, as shown in Figure 8d.

Numerical simulations, however, predict that only 5–7% of polymers and surfactant actually react in the main channel conditions, as shown in Figure 8, before they leave the microchip. The complexation reaction completes in bulk and, therefore, produces complexes with size characteristics typical for macroscopic synthesis.

To minimize after-chip reactions, we proposed a combined approach to the synthesis of polyelectrolyte-surfactant complexes in two-phase microflows. The experimental conditions were set to provide linear aqueous and oil threads, which break-up into droplets before the flows leave the main channel. We used serpentine channels, which both produce linear threads and perform intensive mixing of reagents in droplets.

**Figure 9:**
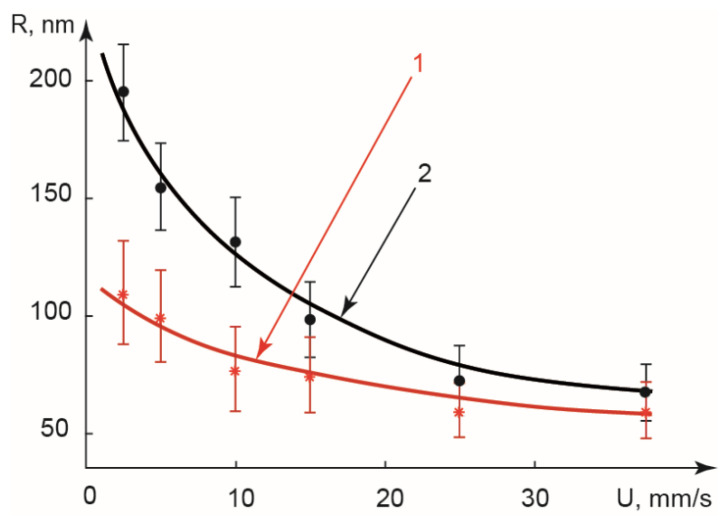
Hydrodynamic radii of PDADMAC-SDS complexes synthesized in serpentine microfluidic threads. Flowrate ratios φ = 1 (curve 1) and φ = 0.3 (curve 2).

PDADMAC-SDS complexes synthesized in the combined droplet-thread mode were characterized by DLS. Figure 9 summarizes the hydrodynamic sizes of complexes synthesized in various conditions of this combined mode.

According to Figure 9, sizes of complexes synthesized at flow velocities above 20 mm/s are 70–90 nm, close to those of complexes produced in small droplets of the droplet-only mode (70–80 nm). It agrees with numerical simulations predicting a small contribution of reactions in threads to the overall product yield in high flow velocity conditions. A predominant yield of the reaction product is provided by reactions in droplets after rapid mixing of the reagents when the droplet break-up occurs near the main channel end.

Lowering flow velocities from 20 mm/s to 2.5 mm/s resulted in growth of complexes from ≈70–80 nm to ≈200 nm, depending on thread widths. In a wider (100 µm) thread (Figure 9, Curve 1), the growth is not so intensive and just slightly exceeds the experimental error. It agrees with numerical simulations, which predict a less intensive diffusion mixing and lower product yield in wider threads. In such experimental conditions, the thread producing Curve 1 is found to be too wide to form a sufficient number of large aggregates and show a significant growth trend.

The narrower thread (Figure 9, Curve 2) demonstrates a considerable growth in the size of complexes at flowrates below 20 mm/s from 70 to 200 nm. The largest size of produced complexes (≈200 nm) exceeds the maximum size of PDADMAC-SDS aggregates synthesized from the same precursor solutions in bulk (≈150 nm). A possible explanation for this effect is that a more intensive diffusion mixing in such threads provides a larger contribution of PDADMAC-SDS complexation products in these threads to the total product yield.

The dispersity of aggregates synthesized in this mode (PDI ≈ 0.2–0.25) is also lower than that of complexes synthesized in bulk (PDI ≈ 0.3–0.4). A possible reason is that the reaction conditions remain ordered in the combined thread-droplet mode (smooth diffusive mixing in threads and homogeneous media in droplets after break-up).

Thus, a combined thread-droplet approach synthesized PDADMAC-SDS complexes in the range of R ≈ 70–200 nm from the same precursor solutions, which exceeded the size range of aggregates synthesized in macroscopic conditions and produced complexes with lower dispersity. Our future research activities will focus on a more detailed analysis of reaction conditions in two-phase flows by fluorescent microscopy, and high-speed imaging and synthesis of drug delivery system components, such as polysaccharide nanoparticles, in the proposed droplet-thread mode.

## 4. Conclusions

Microfluidic two-phase flows provide a variable and controlled environment for the synthesis of polyelectrolyte-surfactant associates with tailored size characteristics and dispersities. PDADMAC-SDS complexes in the size range of R = 70–200 nm and PDI = 0.2–0.25 were synthesized in microchips. Two-phase microfluidic confinement produced polymer-surfactant associates of a broader size range and lower dispersities compared to macroscopic synthesis conditions (R = 100–120 nm and PDI = 0.3–0.4).

The ratio of aqueous and oil phase flowrates and the capillary number are shown to be the priority factors that control formation of polyelectrolyte-surfactant droplets and threads. In droplets, rapid convective mixing is comparable to a characteristic time of PDADMAC-SDS reaction. In threads, mixing is diffusion-controlled and reaction completes after chip. A thread-droplet mode combines different mixing conditions and eliminates undesirable post-chip reactions. It synthesized complexes of the broadest size range and lower dispersities, as compared to other microfluidic alternatives or synthesis in bulk. This approach offers new options for the tailored synthesis of functional polyelectrolyte-surfactant associates suitable for drug delivery applications or fast biochemical analysis in lab-on-chip devices.

## Data Availability

Not applicable.

## Supplementary Materials

The following supporting information can be downloaded at: https://www.mdpi.com/article/10.3390/polym14245480/s1, Figure S1: Geometry of a W-type microfluidic chip with the length L and the width W.
