# Supplementary material for: Tuning Properties of Polyelectrolyte-Surfactant Associates in Two-Phase Microfluidic Flows
Source: Polymers (Basel). 2022 Dec 14;14(24):5480. doi: 10.3390/polym14245480 (PMC9788532; doi:10.3390/polym14245480)
Supplement: Supplementary file 1 [file polymers-14-05480-s001.zip › polymers-2092626-supplementary.pdf]

MATHEMATICAL MODEL OF POLYELECTROLYTE-SURFACTANT COMPLEXATION IN AN AQUEOUS MICROFLUIDIC THREAD

Mathematical Model for a W-type Chip

Figure S1 shows the model of a 3-input microfluidic device used for generating thread of PDADMAC-SDS reactive solutions:

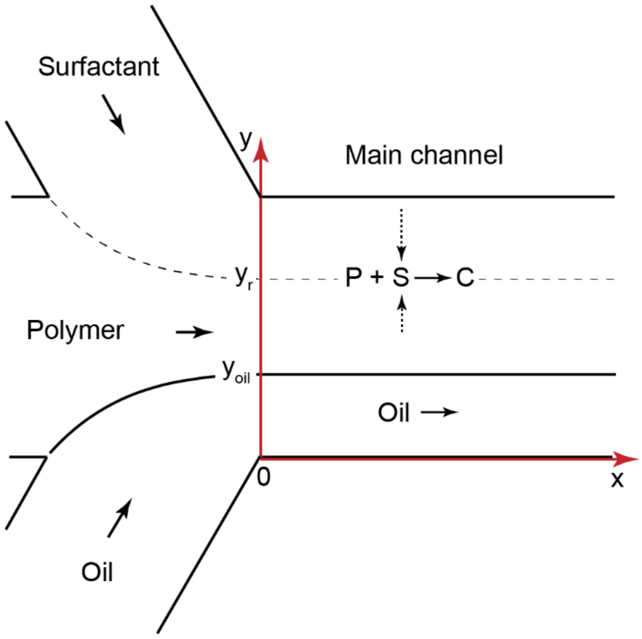

Figure S1. Geometry of a W-type microfluidic chip with the length L and the width W. P – polyelectrolyte, S – surfactant, C – complex, y_{oil} is the boundary of the organic phase flow, y_r is the reaction front coordinate.

Start with a general reaction equation:

$$\mathrm{P} + \mathrm{S} \leftrightarrow \mathrm{C} \tag{1}$$

where P is the concentration of polyelectrolyte ionogenic groups, S is the concentration of surfactant ions, and C is the concentration of polyelectrolyte ionogenic groups with bound surfactant ions.

The rate law for polymer is:

$$[\mathrm{P}]'_{\tau} = -\mathrm{k_f}[\mathrm{P}][\mathrm{S}] \tag{2}$$

where k_f is the association rate constant.

If such a reaction occurs in a microfluidic channel, we need to add the diffusion term $\mathrm{D_P}\Delta[\mathrm{P}]$:

$$[P]'_\tau = D_A \Delta[P] - k_f[P][S] \tag{3}$$

and the convection term $\nabla[P]$:

$$[P]'_\tau + U(y)\nabla[P] = D_A \Delta[P] - k_f[P][S] \tag{4}$$

where D_P is the diffusion coefficient of the polyelectrolyte ionogenic groups (equal to that of polymer macromolecules), U(y) is the flow velocity, ∇ is the Nabla operator Δ is the Laplacian operator, and x is the axial coordinate of a point in a microchannel.

To simplify this equation, consider only the axial convection of a pressure-driven laminar flow in a microchannel, only the radial diffusion of the reacting species and steady state conditions in the microchip:

$$U(y)[P]'_x = D_A[P]''_{yy} - k_f[P][S] \tag{5}$$

where x and y are axial and radial coordinates in the main channel (Fig. 1), $[P]'_x$ and $[P]''_{yy}$ are the partial derivatives of polymer concentration in the main channel.

The Eq. (5) is a non-linear second-order partial differential equation with a reversible reaction as a source term.

Similar equations are derived for surfactant S and the reaction product C, so we obtain a system of partial differential equations that characterize behavior of a reacting polyelectrolyte-surfactant system in a microchannel shown in Fig. 1:

$$\begin{cases} U(y)[P]'_x = D_P[P]''_{yy} - k_f[P][S] \\ U(y)[S]'_x = D_S[S]''_{yy} - k_f[P][S] \\ U(y)[C]'_x = D_C[C]''_{yy} + k_f[P][S]] \end{cases} \tag{6}$$

where [S] is the surfactant concentration, and [C] is the molar concentration of monomer binding sites that bound surfactant ions; D_S, and D_C are the diffusion coefficients of surfactant molecules and polymer-surfactant complexes.

The boundary conditions for the walls of the microchannel and the boundary between aqueous and oil fluid threads are derived from the assumption that the reaction species do not penetrate through them. For polymer:

$$\begin{cases} [P]'_y(y = y_{oil}) = 0 \\ [P]'_y(y = W) = 0 \end{cases} \tag{7}$$

same for the surfactant S and complex C.

The boundary conditions for the junction of the input flows (x = 0) formalize that the concentration of a reagent is equal to the initial concentration in the incoming fluid thread and is zero elsewhere, while the initial product concentration is zero:

$$\begin{cases} [P](x=0,y) = \begin{cases} [P]^0, y_{oil} \le y \le y_r \\ 0, y < y_{oil}, y > y_r \end{cases} \\ [S](x=0,y) = \begin{cases} 0, y < y_r \\ [S]^0, y \ge y_r \end{cases} \\ [C](x=0,y) = 0 \end{cases} \quad (8)$$

The Matlab Script for Convection-Diffusion-Reaction Equation of Polymer-Surfactant Association in Reactive Aqueous Fluid Threads of a Microfluidic Channel

```
%pdex4_Polymer_surf_two-phase
%First set global variables to be used by all the functions in this script:
global W H L Q_pol Q_solv Q_surf Dpol Dmol Dcompl Cpol Csurf kaf kab U k p Z;

acc=500;

k = [1;1;1];
p = [1;2;3];
%INPUT THE VALUES, WHICH CHARACTERIZE THE MICROFLUIDIC SYSTEM AND THE
POLYMER-SURFACTANT SOLUTION:

% Input channel width W, µm; channel height, H, µm; and channel length, L, mm:
W=200; H=100; L=16;
%Input the flowrates of polymer Q_pol, organic solvent Q_solv and surfactant Q_surf, µl/min:
Q_pol = 10; Q_solv = 30; Q_surf = 10;

%Input the diffusion coefficients of polymer, molecular surfactant Dmol,
%micellar surfactant Dmic, and a polymer-surfactant complex Dcompl µm^2/s:
Dpol = 49; Dmol=454; Dcompl = 30;

%Input initial concentration of polymer Cpol, surfactant Csurf and CMC of surfactant, mmol/l:
Cpol=6.1; Csurf=6.1;

Z = Csurf/Cpol;

%Input polymer-surfactant association and dissotiation reaction rate constants kaf l/mol*s and
kab, s^-1:
kaf = 10000; kab = 0.00001*kaf;

%Calculate the flow velocity, mm/s:
U=(Q_pol+Q_solv+Q_surf)/(W*H)*10^6/60;
% Pe = 1./l*(Q_pol+Q_solv+Q_surf)/Dpol;
```

```
%Define the system of the convection-diffusion reaction equations: the second order
%partial differential equations with the source - sink terms (reactions)
%for molecular and micellar surfactant:
a=1;
b=1;
m = 0;

x = linspace(0,a,acc);
l = linspace(0,b,acc);

sol = pdepe(m,@pdex4pde,@pdex4ic,@pdex4bc,x,l);
%Polymer:
u1 = sol(:,:,1);
%Molecular Surfactant:
u2 = sol(:,:,2);
%Complex:
u3 = sol(:,:,3);

figure
surf(u3(:,:))
shading interp
% hcb=colorbar;
% hcb.Location = 'south';
% set(hcb,'YTick',[])
colormap(jet)
view(90,-90)
%axis equal
axis off

figure
surf(u3)
shading interp

% -------------------------------------------------------------
%Define coefficients of the convection-diffusion-reaction equation from the
```

```
%dimensionless numbers:
function [c,f,s] = pdex4pde(x,~,u,DuDx)
global W L Dpol Dmol Dcompl kaf kab U Z Cpol;
%Input the Poiselle flow parabolic profile conditions:
Pois = 2*(1-(2.*x-1)^2);
%Introduce the common coefficient for convection components in all the four equations with the use of global k:

%Input source-sink terms for each equation from rate laws:
s1 = -kaf*Cpol*W*W*Z*0.001.*u(1).*u(2);
s2 = -kaf*Cpol*W*W*0.001.*u(1).*u(2);
s3 = kaf*W*W*Z*0.001*Cpol.*u(1).*u(2);
%finally set the coefficients for all the equations:
%convection term:
c = [1;1;1].*Pois.*U*W*W/L;
%diffusion term:
f = [Dpol; Dmol; Dcompl].*DuDx;
%reaction term:
s = [s1; s2; s3];
end

% Set initial conditions:
function u0 = pdex4ic(x)
global Q_pol Q_solv Q_surf
reaction = (Q_pol+Q_solv)/(Q_pol+Q_solv+Q_surf);
u0 = [x<=reaction; x>=reaction; 0];
end

% Finally set boundary conditions:
function [pl,ql,pr,qr] = pdex4bc(~,ul,~,ur,~)
global k p
pl = ul(p);
ql = k;
pr = ur(p);
qr = k;
end
```
